# Expression and significance of DOK2 in colorectal cancer

**DOI:** 10.3892/ol.2014.2672

**Published:** 2014-11-05

**Authors:** XIANMEI WEN, MUXIU ZHOU, YONG GUO, YANWU ZHU, HONG LI, LU ZHANG, LONG YU, XIAOCHENG WANG, XIAOCHUN PENG

**Affiliations:** 1 Department of Pathology, 161st Central Hospital of the People’s Liberation Army, Wuhan, P.R. China; 2 Department of Pathophysiology, Medical School of Yangtze University, Jingzhou, Hubei, P.R. China

**Keywords:** colorectal cancer, DOK2, biomarker

## Abstract

A reduction in the levels of docking protein 2 (DOK2) expression has previously been reported in lung adenocarcinoma and gastric cancer, indicating that this protein acts as a tumor suppressor in solid tumors. The aim of the current study was to determine the significance of DOK2 in colorectal cancer. The study consisted of 102 patients who underwent curative surgery for colorectal cancer. Histopathological and immunohistochemical analysis of DOK2 protein expression levels was performed in issue samples, and univariate and multivariate analyses were used to investigate the correlation between prognosis and the clinicopathological parameters. DOK2 expression was confirmed in the normal colorectal mucosa tissues, which is consistent with the literature, whereas 34 out of 102 (33.3%) tumor specimens were negative. The results revealed that recurrence was more likely to develop in DOK2(−) patients compared with DOK2(+) patients. The DOK2(−) patients also exhibited a poorer five-year overall survival rate (59.1%) compared with the DOK2(+) group (76.4%; P=0.0328). These results indicate that DOK2 may potentially be used as a marker of poor prognosis in patients with colorectal cancer following curative resection.

## Introduction

Colorectal cancer is the most common type of malignant tumor of the digestive system globally, and its morbidity and mortality are increasing (1). Despite significant progress in the long-term survival of patients, particularly in the early diagnosis and treatment of colorectal cancer, the prognosis of patients with advanced cancer remains poor and heterogeneous (2). The clinicopathological features, including the tumor-node-metastasis (TNM) classification staging system, have certain limitations in assessing the prognosis of the disease due to the considerably variable and heterogeneous nature of colorectal cancer, even between cases at the same stage (3). No biological marker has been generally acknowledged as a cause of the poor prognosis of patients with advanced colorectal cancer (4), although Her2/Neu is regarded as a novel biological marker for breast cancer and is used for guiding the individualized treatment (5–7). Therefore, finding such a marker of colorectal cancer is an urgent requirement.

Docking proteins (DOK)1–3 are adaptor proteins that act in feedback loops to modulate the signaling of tyrosine kinases, including certain tyrosine kinase receptors such as platelet-derived growth factor receptor, epidermal growth factor receptor, c-Kit, Tie2 and Her2/Neu (8). A previous study indicated the clinical significance of DOK2 in evaluating the prognosis of gastric cancer patients (9). However, little is known with regard to the significance of DOK2 in patients with colorectal cancer, another type of gastrointestinal cancer. Accordingly, in the present study, the expression and significance of DOK2 in colorectal cancer was investigated.

## Materials and methods

### Patients

Between October 2005 and March 2007, 102 consecutive patients diagnosed histopathologically with colorectal cancer underwent surgery at the 161st Central Hospital of the People’s Liberation Army (Wuhan, Hubei, China). This study was approved by the ethics committee of Yangtze University (Jingzhou, China). All patients provided informed written consent to participate in this study.

In all patients, pathological stage (pStage) I–III colorectal cancers were newly diagnosed, and no patients had received any chemotherapy or radiotherapy prior to surgery. Subsequent to surgery, patients attended follow-up appointments every three months and underwent appropriate clinical examinations. A total of six frozen samples (N1–N6) were chosen at random from 102 patients to analyze by western blotting, as described later.

### Immunohistochemical staining

DOK2 was detected by immunohistochemical staining, as described previously (10). Briefly, following deparaffinization in xylene and dehydration in graded ethanol solutions, 3.0-μm sections of colorectal cancer tissue and normal colorectal mucosa were heated at 121°C for 20 min in ethylenediaminetetraacetic acid-Tris buffer (pH 9.0) for antigen retrieval. Endogenous peroxidase activity was blocked via incubation of the sections with 30 ml/l hydrogen peroxide for 20 min. The tissue sections were incubated with a primary mouse anti-DOK2 monoclonal antibody (sc-17830; dilution 1:200; Santa Cruz Biotechnology Inc., Santa Cruz, CA, USA) at 4°C overnight, and stained using the labeled streptavidinbiotin method. Negative controls of immunohistochemical reactions were established by omission of the primary antibody. Lymphocytes were used as the positive control. DOK2 staining in each colorectal cancer sample was judged positive when the cancer cells in the section were immunoreactive to DOK2. All slides were assessed independently by two pathologists, and then in consensus in the case of disagreements. The two pathologists were blinded to the clinicopathological data.

### Western blot analysis

Protein extracts from six samples were prepared, and then the protein concentration was determined by the Bradford method (11). SDS sample buffer [6X; 50 mmol/l Tris-HCL (pH 6.8), 100 mmol/l DTT, 2% SDS, 0.1% bromophenol blue and 10% glycerol; Boster Company, Wuhan, China] was added to the extracts prior to denaturing by boiling for 10 min. Equal aliquots (80 μg) of protein were analyzed by 12% SDS-PAGE, followed by electrotransfer onto nitrocellulose membranes (BioAdvantage Co., Ltd., Changsha, China). Following blocking with 10% skimmed milk in Tris-buffered saline containing 0.1% Tween 20 (TBST; Boster Company), the blots were incubated overnight with the primary mouse anti-DOK2 monoclonal antibody (1:1,500; diluted in TBST/4% skimmed milk; Santa Cruz Biotechnology Inc.) at 4°C. Secondary horseradish peroxidase-conjugated goat anti-mouse immunoglobulin G (1:2,000; Jackson Co., Jackson, MS, USA) was applied at room temperature for 45 min. The reactions were developed with the 3,3′-diaminobenzidine kit detection system (Pharmacia, Pittsburgh, PA, USA). β-actin was detected using mouse anti-β-actin monoclonal antibody (Google Biological Co., Ltd., Wuhan, China) as the internal control.

### Statistical analysis

Correlations between DOK2 expression and various clinicopathological parameters were evaluated using the χ^2^ test and Fisher’s exact probability test. Prognostic variables were assessed with a log-rank test, and overall survival (OS) and relapse-free survival (RFS) rates were analyzed using the Kaplan and Meier method. P<0.05 was considered to indicate a statistically significant difference.

## Results

### Immunostaining for DOK2 in normal colorectal mucosa and colorectal cancer tissues by immunohistochemistry

**Figure 1:**
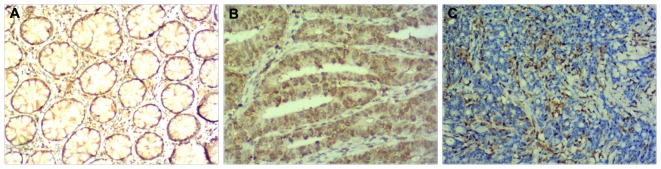
Immunostaining for DOK2 in normal colorectal mucosa and colorectal cancer tissues. (A) Immunoreactivity to DOK2 in the normal colorectal mucosa, (B) DOK2 immunoreactivity in colorectal adenocarcinoma diagnosed as moderately-differentiated adenocarcinoma, and (C) negative immunostaining for DOK2 in colorectal adenocarcinoma diagnosed as poorly-differentiated adenocarcinoma (magnification, ×100). DOK2, docking protein 2.

The normal colorectal mucosa and colorectal cancer tissues were evaluated for immunoreactive DOK2 with specific antibodies. DOK2 was detected in all normal colorectal mucosa samples (100%), and the intensity of immunostaining for DOK2 was strong in the mucosal epithelial cells (Fig. 1A). A total of 68 out of 102 patients (66.7%) were diagnosed with moderately-differentiated adenocarcinoma, and immunoreactivity was also detected in the colorectal cancer tissue samples of these patients; however, it was weaker than that of normal tissues (Fig. 1B). No DOK2 immunoreactivity was observed in the remaining samples from the 34 patients (33.3%) with poorly-differentiated adenocarcinoma. Immunoreactivity to DOK2 in lymphocytes was used as a positive control (Fig. 1C).

### Expression of DOK2 protein in colorectal cancer, as detected by western blotting

**Figure 2:**
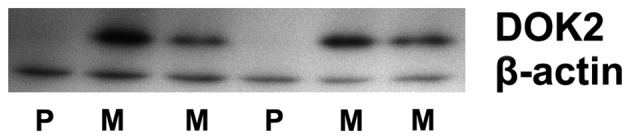
Western blotting to detect DOK2 protein expression. Six samples were selected, four DOK-2(+) cases and two DOK2(−) cases. DOK2, docking protein 2; P, poorly-differentiated adenocarcinoma; M, moderately-differentiated adenocarcinoma.

The results of the western blot analysis were consistent with the results of the immunohistochemical staining. The two samples of poorly-differentiated adenocarcinoma were negative for DOK2 expression, however, the expression level in the four samples of moderately-differentiated adenocarcinoma was high (Fig. 2).

### Correlation between DOK2 expression and clinicopathological parameters

The correlations between DOK2 expression and various clinicopathological parameters are listed in Table I. In the colorectal cancer samples, the differentiated-type tumors (including papillary, and well- and moderately-differentiated adenocarcinomas), based on histopathological grade, were significantly more likely to be DOK2(−) than DOK2(+) [DOK2(−), 69.7%; DOK2(+), 30.3%; P=0.001]. No significant difference in clinical characteristics (age, gender, vascular invasion, localization of the cancers and TNM stage; Fisher’s exact test; P>0.05) was identified.

### Correlation between DOK2 expression and clinical outcome

**Figure 3:**
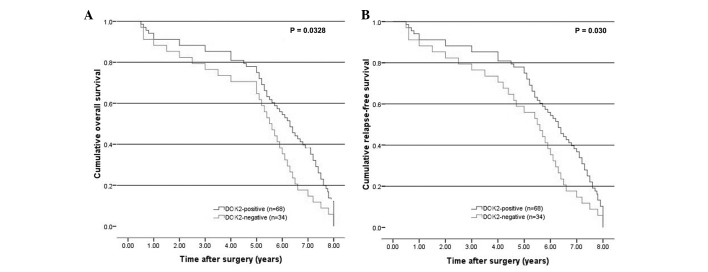
Kaplan-Meier curves for overall survival (OS) and relapse-free survival (RFS) according to DOK2 expression. (A) OS curve and (B) RFS curve according to DOK2 expression for all patients. Differences between the two groups were evaluated by log-rank test. Ordinate survival rate, abscissa time after surgery (years). DOK2, docking protein 2.

Post-surgery disease relapse was diagnosed in 27 out of 102 patients (26.5%), with a median time to relapse of 17.8 months. The relapse rate in the DOK2(−) patients was significantly higher than that in the patients with DOK2(+) tumors (P=0.043). The OS and RFS rates were significantly poorer for the patients with DOK2(−) tumors than for DOK2(+) patients [five-year OS: DOK2(−), 59.1%; DOK2(+), 76.4%; P=0.0328; and five-year RFS: DOK2(−), 58.1%; DOK2(+), 73.0%; P=0.030] (Fig. 3).

## Discussion

Colorectal cancer is the most common type of malignant neoplasm and the third leading cause of cancer-related mortality among males and females (12). Currently the five-year survival rate of colorectal cancer is ≥50%; the rate for early-stage cancer is ~80% and the rate for advanced-stage cancer is ~30%. The discovery of a tumor marker for the early diagnosis of colorectal cancer would improve the 5-year patient survival rate. In fact, the majority of patients are in the advanced stages of the disease when they seek treatment (13). Recurrence and metastasis are primarily associated with this subset of patients (14). There is increasing demand for the identification of a potential biomarker to predict the presence and recurrence of tumors, particularly when the best chance of a successful treatment requires the early diagnosis and timely surgery of a tumor that is already malignant, but not yet invasive, such as colorectal cancer (15). Growing evidence indicates that DOK2 may be a promising tumor biomarker. It has been found that the loss of DOK2 accelerates lung tumorigenesis in genetically engineered mouse models (16). DOK2 has been identified as a tumor suppressor gene in a number of types of tumors, including gastric adenocarcinoma and acute leukemias (9,17), however, little has been reported with regard to DOK2 in colorectal cancer. In the current study, the results demonstrated that the expression of DOK2 was downregulated in 34 colorectal cancer tissue samples from patients with poorly-differentiated adenocarcinoma, while it was expressed in all of the normal colorectal tissues, suggesting that DOK2 may be involved in the initiation and progression of colorectal cancer, in addition to the prognostic assessment. To further evaluate the clinical significance of the reduced DOK2 expression in colorectal cancer, the association between DOK2 expression and clinicopathological parameters was investigated. The results showed that the negative expression of DOK2 was significantly more likely to be observed in the well- and moderately-differentiated adenocarcinomas than positive DOK2 expression.

It has been established that the prediction of recurrence and metastasis following curative resection may allow the determination of the requirement for intensive follow-up and adjuvant therapy (18). To determine the correlation between DOK2 expression and the clinical outcome, further clinicopathological analyses were performed in the present study. The results revealed and poorer OS rates and a significantly high risk of relapse in patients with DOK2(−) tumors compared with those with DOK2(+) tumors. These data indicate that DOK2 may be a significant prognostic indicator in colorectal cancer. Notably, K-ras gene-targeted therapy has begun to be examined as a promising treatment in clinical trials (19,20). Similarly, DOK2 is the next potential therapeutic target for colorectal cancer treatment, and will undoubtedly yield huge benefits to patients with advanced or recurrent and metastatic colorectal cancer. However, further studies are required to elucidate the potential mechanisms by which low DOK2 expression results in a poor prognosis.

In conclusion, the present study demonstrated that DOK2 is expressed in the normal gastric mucosa and 66.7% of colorectal cancer samples. However, it was not detected in samples from patients with poorly-differentiated adenocarcinoma. These results indicate a potential use for DOK2 as a marker for the prediction of prognosis for patients with colorectal cancer following curative resection, which may provide a novel therapeutic target for colorectal cancer.

## Figures and Tables

**Table I:** Correlation between DOK2 expression and various clinicopathological parameters.

		DOK2 expression	
		
Parameters	P-value	Positive	Negative
All cases	68	34	
Age (<66/>66 years)	30/38	20/14	0.208
Gender (female/male)	25/43	12/22	1.000
Differentiation (poor/well and moderate)	58/10	11/23	0.001
pT (T3–4/T1–2)	42/26	21/13	1.000
pN (N3–4/N0–1)	27/41	10/24	0.384
pStage (I/II/III)	20/23/25	10/11/13	0.986
Lymphatic infiltration (positive/negative)	54/14	28/6	0.797
Venous invasion (positive/negative)	31/37	15/19	0.888
Relapse cases (n=27)	13	14	0.031
Lymph node (n=8)	6	2	0.630
Peritoneum (n=9)	6	3	1.000
Hematogenous (n=17)	7	10	0.015
Liver (n=11)	3	8	0.005

DOK2, docking protein 2; pT, pathological tumor stage; pN, pathological nodal stage.
